# Comparability of Objective Structured Clinical Examinations (OSCEs) and Written Tests for Assessing Medical School Students’ Competencies: A Scoping Review

**DOI:** 10.1177/01632787231165797

**Published:** 2023-03-23

**Authors:** Oswin Chang, Anne M. Holbrook, Simran Lohit, Jiawen Deng, Janice Xu, Munil Lee, Alan Cheng

**Affiliations:** 1 Clinical Pharmacology and Toxicology Research, St Joseph’s Healthcare Hamilton; 2 Faculty of Health Sciences, 62703 McMaster University; 3 Division of Clinical Pharmacology and Toxicology, 25479 McMaster University; 4 70384 Schulich School of Medicine and Dentistry,University of Western Ontario

**Keywords:** clinical competence, curriculum, COVID-19, distance education, professional education

## Abstract

Objective Structured Clinical Examinations (OSCEs) and written tests are commonly used to assess health professional students, but it remains unclear whether the additional human resources and expenses required for OSCEs, both in-person and online, are worthwhile for assessing competencies. This scoping review summarized literature identified by searching MEDLINE and EMBASE comparing 1) OSCEs and written tests and 2) in-person and online OSCEs, for assessing health professional trainees’ competencies. For Q1, 21 studies satisfied inclusion criteria. The most examined health profession was medical trainees (19, 90.5%), the comparison was most frequently OSCEs versus multiple-choice questions (MCQs) (18, 85.7%), and 18 (87.5%) examined the same competency domain. Most (77.5%) total score correlation coefficients between testing methods were weak (*r* < 0.40). For Q2, 13 articles were included. In-person and online OSCEs were most used for medical trainees (9, 69.2%), checklists were the most prevalent evaluation scheme (7, 63.6%), and 14/17 overall score comparisons were not statistically significantly different. Generally low correlations exist between MCQ and OSCE scores, providing insufficient evidence as to whether OSCEs provide sufficient value to be worth their additional cost. Online OSCEs may be a viable alternative to in-person OSCEs for certain competencies where technical challenges can be met.

## Introduction

Multiple-choice questions (MCQs), written answer questions, and Objective Structured Clinical Examinations (OSCEs) form the current backbone of medical and other health professional education assessments internationally. In Canada, trainees are usually evaluated using the CanMEDS framework with its seven domains—Medical Expert, Communicator, Collaborator, Leader, Health Advocate, Scholar, and Professional (Royal College of Physicians and Surgeons of Canada, 2015). Other countries have a similar set of core competencies that are organized within their own respective framework names (Accreditation Council for Graduate Medical Education, 2022; General Medical Council, 2017; Medical Board of Australia, 2021).

MCQs with single best answer have become the dominant testing method but have well-known limitations – mainly the cueing effect, testing the ability to recognize an answer rather than to generate an answer, and difficulty representing many important areas of medicine where no appropriate distractors can be created (Sam et al., 2016). Long answer written questions are used sparingly as they are time-consuming to mark.

OSCEs were first developed in the 1970s to improve the assessment of clinical skills by creating a more objective setting in which examiners and patients could be standardized so that all candidates would be assessed in a similar way to reduce bias (Harden et al., 1975). Since its advent, OSCEs have been incorporated into all medical curricula and most certification requirements in Canada, the United States, and many countries abroad (Accreditation Council for Graduate Medical Education, 2022; Australian Medical Council Limited, 2021; General Medical Council, 2023; Medical Council of Canada, 2023). OSCEs have been found to be useful in preparing students to manage conditions both common and rare as well as perform interventions while incorporating competency domains (Dizon et al., 2021; Jutant et al., 2022; Pugh et al., 2015).

Understanding the relative merits of both testing methods in assessing health professional trainees is necessary to ensure resources, including scarce faculty, staff, and simulated patient time and costs are used wisely. Costs associated with running OSCEs can easily surpass $600 USD per student, with some institutions spending upwards of $900 USD per student (Grand’Maison et al., 1992; Sudan et al., 2015; Walsh et al., 2009) with extensive time commitments to recruit, train, set up, equip, operate, and clean up. Written testing methods do not require the same time and resources, so they are the logical assessment method unless OSCEs can be shown to be superior for testing certain competencies.

During the COVID-19 pandemic when in-person OSCE assessments were not possible, many health professional schools rapidly implemented online OSCEs as a substitute (Hytönen et al., 2021; Jamil et al., 2022; Luke et al., 2021; Nusanti et al., 2021). While online OSCEs are modeled after in-person ones, the online format itself is a major change, and the number of stations or competencies tested may be altered; therefore, its validity and reliability are uncertain.

Our effort to develop efficient assessment methods for Clinical Pharmacology and Toxicology (CPT) curricula in medical education with its focus on therapeutics and toxicology knowledge, patient communication, and safe prescribing in a rapidly evolving discipline, led to the importance of evaluating the relative merits of OSCEs versus written exams or online OSCEs (Holbrook et al., 2019; Liu et al., 2018; Qayyum et al., 2012; Wu et al., 2015; Zhang et al., 2019). Understanding the value of various testing methods is an important undertaking for every health professional training program worldwide with limited resources.

The objective for this scoping review was to characterize the evidence on the comparability of OSCEs versus written exams and in-person versus online OSCEs for assessing competencies of health professional trainees.

## Method

The protocol for this study was registered on OSF registries (https://osf.io/6u8fy/) (Holbrook et al., 2021). We conducted this scoping review in accordance with the Preferred Reporting Items for Systematic Reviews and Meta-Analyses Extension for Scoping Reviews (PRISMA-ScR) framework (Tricco et al., 2018).

### Research questions

The first research question is as follows: Are OSCEs a comparable evaluation method to multiple-choice or short/long answer written questions in assessing health professional trainees and, if so, for which competency domains (these seven competency domains were used: Medical Expert – the integrating role, Communicator, Collaborator, Leader, Health Advocate, Scholar, and Professional)?

The second research question asked: Are online OSCEs (examinee and examiner, with or without simulated patient, all online and at distance from each other) superior to in-person OSCEs (examinee and examiner, with or without simulated patient in the same location) in terms of logistics, feasibility, resource intensity, for assessing health professional trainees, and if so, for which competency domains?

### Eligibility criteria

Our inclusion criteria are highlighted as follows within the PICOTS framework (Guyatt et al., 2015). For Q1 (OSCEs vs. written tests), the **Population** was health professional trainees (e.g., students or residents), excluding veterinary trainees; the **Intervention** was OSCE stations; the **Comparator** was written or typed testing methods (e.g., multiple-choice questions, short/long form answers); the **Outcomes** were overall score comparisons and individual competency domain score comparisons, as measured by the study itself; the **Timeframe** included studies published between 1946 and 2021; and the **Study design/type** included studies that had to have a control/comparison group (e.g., RCTs, cohort studies, case–control studies, historical controls). Included studies also had to report primary data and be written in English.

For Q2 (online vs. in-person OSCEs), the **Population** was health professional trainees (e.g., students or residents), excluding veterinary trainees; the **Intervention** was online OSCE stations (test taker communicates information through an online platform); the **Comparator** was in-person OSCE stations (test taker communicates information in a face-to-face, in-person setting); the **Outcomes** were overall score comparisons, individual competency domain score comparisons, and logistics, feasibility, and resource intensity, as measured by the study itself; the **Timeframe** included studies published between 2000 and 2021 (timeframe was reduced as online OSCEs are a recent phenomenon); and the **Study design/type** included studies that had to have a control/comparison group (e.g., RCTs, cohort studies, case–control studies, historical controls). Included studies also had to report primary data and be written in English. For both research questions, editorials, commentaries, and guidelines were excluded. Conference abstracts were considered since they may have relevant information or data.

### Literature search

We conducted searches in Ovid MEDLINE (Epub Ahead of Print, In-Process, In-Data-Review and Other Non-Indexed Citations and Daily, Ovid MEDLINE(R) Daily and Ovid MEDLINE(R) 1946 to Present) and Ovid EMBASE. The search strategy was created with the assistance of university and hospital research librarians and can be found in Supplemental Appendix 1.

We searched databases from inception (1946 for MEDLINE and 1974 for EMBASE) to August 3, 2021 for Q1, and from 2000 to August 8, 2021 for Q2. No restrictions were placed for a study’s country of origin. Only these two bibliographic databases were searched given the time-sensitive nature of the study and because there is evidence that searching MEDLINE and EMBASE provides a sufficient proportion of relevant studies (93%) compared to searching all databases (Bramer et al., 2017; Rice et al., 2016; van Enst et al., 2014).

### Study selection

#### Screening

Several authors performed title and abstract screening in duplicate and independently on the online platform Covidence (https://www.covidence.org/). Disagreements in screening were resolved by consensus. Studies that passed initial screening underwent full-text screening in Covidence (Supplemental Appendix 2, which exhibits process flow). We conducted full-text screening in the same manner and recorded reasons for exclusion.

#### Data extraction

Paired and independent data extraction was completed using Microsoft Excel. A standardized form was used by the team to extract data from the studies. We extracted information on study characteristics (e.g., publication year, study design, setting, sample size), participant characteristics (e.g., health profession, level of education/training), assessment outcomes including competency domains, and statistics on the results. In anticipation of varying competency domain descriptors used in different countries, each domain description was reconciled to a CanMEDS domain for consistency. For example, if an OSCE station assessed a trainee’s ability to provide patient-friendly education, this station would be classified as assessing the Communicator domain. As this study was a scoping review, a formal risk of bias assessment was not performed (Peters et al., 2015).

#### Data synthesis

The analyses were descriptive and reported on 1) comparisons between OSCEs and written testing methods (through total scores and competency domain scores), and 2) comparisons between in-person and online OSCEs (through total scores and competency domain scores).

## Results

### Q1 – OSCEs versus written testing methods

**Figure 1.**
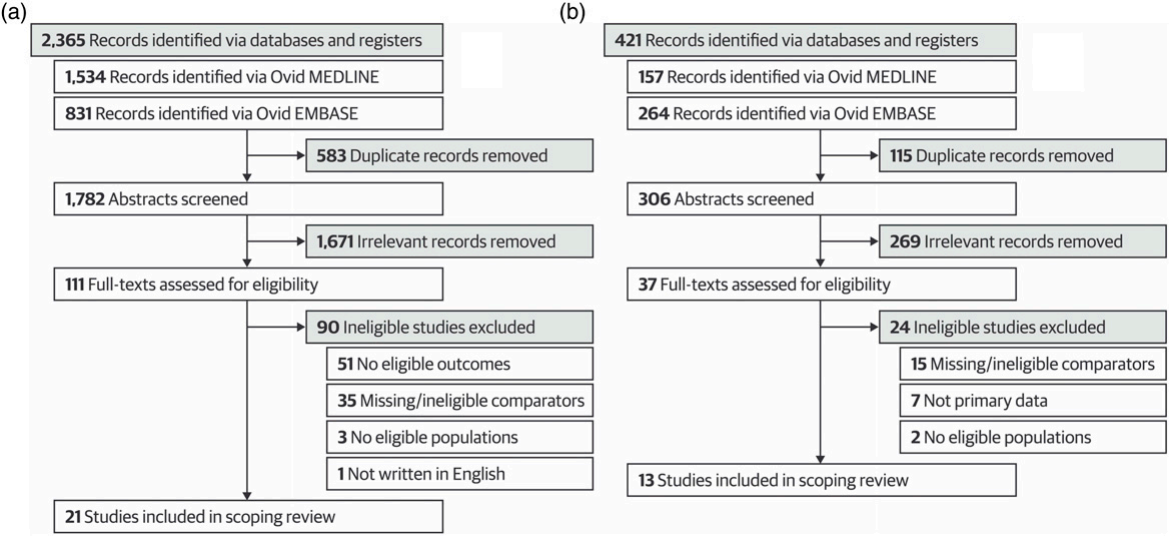
Preferred Reporting Items for Systematic Reviews and Meta-Analyses Extension for Scoping Reviews (PRISMA-ScR) flowchart for the identification and selection of studies comparing a) Objective Structured Clinical Examinations (OSCEs) with written testing methods and b) in-person and online OSCEs.

The search yielded 1782 eligible citations, with 21 relevant articles remaining after title, abstract, and full-text screening (Andrades et al., 2017; Auewarakul et al., 2005; Butler et al., 2017; Chibnall & Blaskiewicz, 2008; Couto et al., 2019; Dennehy et al., 2008; Dong et al., 2014, 2017; Eftekhar et al., 2012; Gillette et al., 2017; Gilson et al., 1998; Hull et al., 1995; Huwendiek et al., 2017; Jameel et al., 2015; Kelly et al., 2013; Nuovo et al., 2006; Schleicher et al., 2017; Schoeman & Chandratilake, 2012; Schwartz et al., 1995; Simon et al., 2002, 2007). The PRISMA flow diagram for this research question can be found in Figure 1 (Page et al., 2021). Detailed study characteristics are shown in Table 1. No studies used a randomized trial (including a randomized crossover) design, but 19 studies employed a weak before-and-after study design, with one each of prediction analysis and cross-sectional study. None ensured that outcome assessors were blinded to group allocation during grading. Twelve of the 21 studies were conducted in the United States, and the remaining were conducted in other countries (e.g., Iran, Pakistan, Thailand). Medical trainees were the focus of 19 studies, and 16 examined students (rather than residents or fellows). The most common written comparison method was MCQs, which were used in 18 studies, and the mean sample size was 258.1 (SD 196.4). More than 4645 participants were included, with no sample size information given by three studies. Characteristics of individual studies can be found in Supplemental Appendix 3.

**Table 1.** OSCEs versus Written Assessments – Study Characteristics and Summary Results.

Study Characteristics	All Studies *N* = 21 (100%)
Publication year	
2015 and onwards	8 (38.1)
Before 2015	13 (61.9)
Country of origin of participants	
USA	12 (57.1)
Other (Brazil, Britain, Germany, Iran, Ireland, Pakistan, Thailand)	9 (42.9)
Study design	
Before-and-after	19 (90.5)
Other	2 (9.5)
Number of OSCE stations^a^	
<10	6 (37.5)
10-19	7 (43.8)
>19	3 (18.8)
In-person OSCE stations^a^	20 (100.0)
Outcome assessors blinded to group allocation during grading	0 (0.0)
Single medical school/training program	20 (95.2)
Type of health profession	
Medicine	19 (90.5)
Dentistry	1 (4.8)
Pharmacy	1 (4.8)
Level of training	
Student	16 (76.2)
Resident	5 (23.8)
Written comparison method(s)^b^	
Multiple-choice question	18 (85.7)
Short answer	4 (19.0)
Long answer	3 (14.3)
Total participant size at beginning of study^a^	
<100	5 (27.8)
100-199	4 (22.2)
200-299	0 (0.00)
300-399	5 (27.8)
>399	4 (22.2)
**Summary results**	**Number (%) studies**
Studies assessing each competency domain	
Medical Expert	18 (85.7)
Communicator	15 (71.4)
Collaborator	0 (0.0)
Leader	0 (0.0)
Health Advocate	0 (0.0)
Scholar	0 (0.0)
Professional	8 (38.1)
Unclear	1 (4.8)
OSCE evaluation scheme^a^	
Checklist	8 (61.5)
Rubric	1 (7.7)
Other	4 (30.8)
Same competency domains compared^a,c^	18 (85.7)
Total score correlation coefficient magnitude^a^: n comparisons with written assessments	
>0.79	0 (0.0)
0.40–0.79	9 (22.5)
<0.40	31 (77.5)
Competency domain score correlation coefficient magnitude^a,d^: n comparisons with written assessments	
>0.79	0 (0.0)
0.40–0.79	2 (6.5)
<0.40	29 (93.5)
‘Gold standard’ comparison included^a,e^	10 (66.7)

*Notes.* OSCE, Observed Structured Clinical Examination;

^a^Information not available for all studies;

^b^More than one answer possible;

^c^Assessments all compared Medical Expert;

^d^Competency domain score included 25 Medical Expert, four Communication, two Professionalism;

^e^e.g., national licensing exams or in-training exams.

The Medical Expert and Communicator roles were most commonly assessed, with 18 and 15 studies, respectively. No studies examined the Collaborator, Leader, Health Advocate, or Scholar roles. A majority of studies used an OSCE checklist as the evaluation method and 10 (66.7%) had a ‘gold standard’ comparison (e.g., national licensing exam or in-training exam). Analyses were based on correlation methods. Of the 40 total score correlations between OSCEs and written tests, 31 comparisons had a correlation coefficient (*r*) magnitude less than 0.40, indicating low correlation. Similarly, 29 of the 31 competency domain score correlations between testing methods yielded an *r* value less than 0.40. In 18 studies, the same competency domains were compared, but only 14 also reported total score correlations (a total of 32 comparisons of which 25 were low (*r* < 0.40)) (Andrades et al., 2017; Auewarakul et al., 2005; Butler et al., 2017; Chibnall & Blaskiewicz, 2008; Couto et al., 2019; Dennehy et al., 2008; Dong et al., 2014, 2017; Eftekhar et al., 2012; Gilson et al., 1998; Hull et al., 1995; Huwendiek et al., 2017; Kelly et al., 2013; Nuovo et al., 2006; Schleicher et al., 2017; Schwartz et al., 1995; Simon et al., 2002, 2007; Schoeman & Chandratilake, 2012). Of the four studies where the Communicator competency domain was compared to written assessments, all correlations were low (Gillette et al., 2017; Kelly et al., 2013; Simon et al., 2002, 2007). Detailed results are reported in Table 1.

### Q2 – in-person versus online OSCEs

Our search yielded 306 potentially relevant articles with 13 articles remaining after title, abstract, and full-text screening (Arrogante et al., 2021; Biolik et al., 2018; Boardman et al., 2021; Darr et al., 2021; Farrell et al., 2021; Hsia et al., 2021; Lara et al., 2020; Martinez et al., 2020; Nackman et al., 2006; Novack et al., 2002; Oliven et al., 2011; Scoular et al., 2021; Sihombing et al., 2021). The PRISMA flow diagram for this research question can be found in Figure 1 (Page et al., 2021). Ten of 13 included studies used a weak before-and-after study design, two were randomized crossover trials, and one was a prospective cohort with concurrent control. No outcome assessors were blinded to group allocation during grading. Nine studies were conducted in the United States, and the remaining four were carried out in Germany, Indonesia, Israel, and Spain. Nine studies focused on medical trainees, and 11 studies examined students (as opposed to residents or fellows). Most included four or more OSCE stations for online and in-person formats, and the mean sample size was 158.8 (SD 84.4). A total of 2064 trainees participated, but two studies reported only the number of trainees who participated in the online OSCE. Full characteristics of included studies can be found in Table 2, and characteristics of individual studies can be found in Supplemental Appendix 4.

**Table 2.** Online versus In-person OSCEs – Study Characteristics and Summary Results.

Study Characteristics	All Studies *N* = 13 (100%)
Publication year	
2021	7 (53.8)
Before 2021	6 (46.2)
Country of origin of participants	
USA	9 (69.2)
Other (Spain, Germany, Israel, Indonesia)	4 (30.8)
Study design	
Randomized crossover trial	2 (15.4)
Prospective cohort with concurrent control	1 (7.7)
Before-and-after	10 (76.9)
Number of in-person OSCE stations^a^	
<4	3 (20.0)
4-6	2 (13.3)
>6	2 (13.3)
Number of online OSCE stations^a^	
<4	4 (40.0)
4-6	3 (30.0)
>6	3 (30.0)
Outcome assessors were blinded to group allocation during grading	0 (0.0)
Single medical school/training program	12 (92.3)
Type of health profession	
Medicine	9 (69.2)
Nursing	1 (7.7)
Pharmacy	3 (23.1)
Level of training	
Student	11 (84.6)
Resident	2 (15.4)
Online OSCEs have separate examiner, examinee, and SP: Yes	8 (46.7)
Total participant size at beginning of study (in-person and online)^a^	
<50	1 (9.1)
50-99	0 (0.0)
100-149	2 (18.2)
150-200	3 (27.3)
>200	5 (45.5)
**Summary results**	**Number (%) studies**
Studies assessing each competency domain	
Medical Expert	9 (69.2)
Communicator	9 (69.2)
Collaborator	2 (15.4)
Leader	0 (0.0)
Health Advocate	2 (15.4)
Scholar	0 (0.0)
Professional	3 (23.1)
Unclear	1 (7.7)
Evaluation scheme^a^	
Checklist	7 (63.6)
Rubric	2 (18.2)
Other	2 (18.2)
Overall score: n comparisons	
No statistically significant differences	14 (82.4)
In-person statistically significantly higher	0 (0.0)
Online statistically significantly higher	3 (17.6)
Medical expert competency domain score^b^: n comparisons	
No statistically significant differences	8 (66.7)
In-person statistically significantly higher	3 (25.0)
Online statistically significantly higher	1 (8.3)
Communication competency domain score: n comparisons	
No statistically significant differences	10 (55.6)
In-person statistically significantly higher	3 (16.7)
Online statistically significantly higher	5 (27.8)
Collaborator competency domain score: n comparisons	
No statistically significant differences	1 (50.0)
In-person statistically significantly higher	1 (50.0)
Online statistically significantly higher	0 (0.0)
Health Advocate competency domain score: n comparisons	
No statistically significant differences	1 (50.0)
In-person statistically significantly higher	1 (50.0)
Online statistically significantly higher	0 (0.0)
Professional competency domain score: n comparisons	
No statistically significant differences	2 (50.0)
In-person statistically significantly higher	2 (50.0)
Online statistically significantly higher	0 (0.0)

*Notes.* OSCE, Objective Structured Clinical Examination; SP, standardized patient;

^a^Information not available for all studies;

^b^No studies addressed the Leader or Scholar role.

The most commonly assessed competency domains were Medical Expert and Communicator, with nine studies each. None of the studies assessed the Leader or Scholar roles. Most studies also used an OSCE checklist as the evaluation method. Of the 17 comparisons of an overall OSCE score derived from summation of individual station scores, 14 of them found no statistically significant difference between in-person and online OSCEs, while three found online OSCE scores were statistically significantly higher. When breaking down scores by competency domains, most studies also found no difference between scores. However, the Communicator competency domain was the most varied, where 10 comparisons found no difference, three found in-person scores were higher, and five found online scores were higher. For the Professional competency domain, two comparisons found no score differences between in-person and online OSCEs, while two found in-person scores to be higher. Overall, it appears that online OSCEs and in-person OSCEs are comparable for assessing the Communicator and Medical Expert competency domains. Results are summarized in more detail in Table 2.

## Discussion

OSCEs have been a standard and popular health professional assessment method for several decades now, particularly for clinical skills, despite the large cost of OSCEs due to the extensive faculty organization and involvement required (Rushforth, 2007; Walsh et al., 2009; Zayyan, 2011). It was therefore surprising not to find any systematic or scoping reviews assessing OSCE comparability to written methods for the assessment of health professional trainee competencies in our searches. Two previous literature reviews did report generally low to moderate correlations between testing methods but focused more on the validity and reliability of OSCEs rather than comparability (Alunno et al., 2020; Walsh et al., 2009). Alunno et al. (2020) reviewed 36 studies on the assessment of resident competencies, finding low score correlation with MCQs but good inter-rater reliability and internal consistency. In contrast, Walsh et al. (2009) found that OSCEs had been inadequately studied for nursing competencies, including their expense, which was prohibitive in some studies.

For our research question on OSCEs versus written testing methods, we found most total score and competency domain score correlations between the formats were low. A previous review found similar results in that most correlation coefficients between standardized patient (SP)-based tests and other measures, including MCQs, short answer tests, clinical ratings, and National Board of Medical Examiners tests were ≤ 0.50 (Turner & Dankoski, 2008; van der Vleuten & Swanson, 1990). Low correlations between the testing methods may not be surprising if OSCEs are truly testing different constructs than written exams or, given the commonly used before-after designs, the two types of assessments are completed at different phases of trainee expertise. Alternatively, it has been suggested that low correlations arise from limited OSCE validity (Turner & Dankoski, 2008). For example, one study had lay volunteers review a total of 100 medical student encounters and found the volunteers’ perceptions of effective communication were not well correlated with OSCE communication checklist scores completed by trained raters in four of five stations (r = 0.03, 0.02, 0.13, and 0.33) (Mazor et al., 2005). An analysis of various OSCE communication checklists found their psychometric properties to be lacking or uncertain in many areas (e.g., content validity, agreement, criterion validity, responsiveness, etc.) (Cömert et al., 2016). These shortcomings are not limited to communication OSCEs and may be an indication of the general problems related to studies on the development and psychometric testing of OSCEs, such as missing data and absence of structure in reporting (Patrício et al., 2009).

Some of the best evidence on the value of OSCEs and written exams come from two studies that correlated exam results to later quality of care. Both certification exams (involving OSCEs and written components) for family medicine in Quebec and the Medical Council of Canada Qualifying Examinations (MCCQE) (written exam) were predictive of the quality of preventative care and disease management during the subsequent first decade of practice (Tamblyn et al., 2002). The second study found that low scores on Part I (written components) of the MCCQE predicted subpar quality-of-care in regulatory college peer assessments and inclusion of Part II results (OSCEs) did not statistically significantly improve predictive power (Wenghofer et al., 2009). These findings indicate a need for further investigation into the added value of including OSCEs in examinations.

The overall consensus from the 13 included studies indicated that online OSCEs were comparable to in-person ones in ability to assess the Medical Expert and Communicator competency domains. In particular, the two studies that employed the most robust study designs (randomized crossover trials) found no statistically significant differences in scores between OSCE formats, providing stronger evidence for their comparability (Biolik et al., 2018; Oliven et al., 2011). However, due to the lack of or small number of comparisons for the other competencies (e.g., Collaborator, Leader, Health Advocate, Scholar, and Professional), more studies will need to be conducted before a definitive conclusion can be reached as to whether online OSCEs are comparable to in-person ones for assessing these domains. Considering that online OSCEs were not widely used pre-pandemic, this rapid transition to online testing has been remarkable, given the negative impact that the pandemic has had on in-person learning. Online OSCEs have also been well-received by trainees, faculty, as well as SPs (Farrell et al., 2021; Hsia et al., 2021; Kelly et al., 2022; Martinez et al., 2020). Thus, online OSCEs seem to be a viable alternative for in-person ones for many station types. However, the advantages in terms of convenience and social distancing are counteracted by the technical difficulties of running an online OSCE as well as the lack of ability to fully test physical exam skills (Kupfer, 2020). The format of choice may ultimately rest upon which competencies are to be evaluated and the availability of in-person faculty evaluators and SPs versus the technical infrastructure and troubleshooting required for an online format. Unfortunately, we are unable to comment on relative costs, logistics, or feasibility, as no data were provided in the studies.

### Strengths and limitations

This present study has several strengths. To our knowledge, this study is the first scoping review to summarize evidence on OSCEs compared to written tests as well as to compare in-person and online OSCEs for assessing clinical competence in health professional trainees. Moreover, we were able to gather enough evidence to provide an evaluation for more than one competency domain. However, there are limitations to our scoping review being able to come to strong conclusions. One is the lack of high-quality studies investigating the assessment abilities of OSCEs. Based on the design of the studies, the quality of evidence is low, but as this was a scoping review, we did not formally assess the included studies’ risk of bias. Moreover, articles not published in English were not included, so it is possible that a small number of relevant studies were missed. Similarly, searching more exhaustively in other bibliographic databases might have identified a few additional articles.

### Implications

Based on the findings of this review, we decided that there was insufficient evidence to support the superiority of OSCEs in assessing competencies compared to written exams. As the initial impetus of the study was to determine whether to mount a CPT OSCE – either in-person or online, the focus of our larger study has shifted towards developing and validating computerized marking of prescription writing. We have a severe shortage of faculty to supervise OSCEs, and written exams, while limited for some competencies, are more feasible at this time. Other health professional education programs will continue to assess on a regular basis whether they require OSCEs, either in-person or online, depending on their faculty and financial resources.

Future studies should compare in-person OSCEs, online OSCEs, and written testing methods for the same competency domains, using concurrent comparison groups. Moreover, the validity and reliability of OSCEs require further work, although this undertaking would require the difficult job of producing a ‘diagnostic standard’ for threshold of competency beyond expert judgment. Additionally, future explorations comparing in-person and online OSCEs should examine other competency domains besides the Medical Expert and Communicator roles in order to provide evidence for comparability in testing the other competencies. Greater focus should also be placed on understanding the logistics, costs, and feasibility of OSCEs.

## Conclusion

This scoping review was unable to confirm the value for additional resources and costs required for OSCEs compared to written exams. We found generally low correlations between scores on written assessments and OSCEs for medical trainees for different competency domains. Online OSCEs appear to be comparable to in-person OSCEs in assessing the Medical Expert and Communicator roles, although no conclusion could be arrived at for the remaining competencies due to a lack of data. These results indicate the need for further research to understand why correlations were low between testing methods and whether there are competency domains for which OSCEs provide sufficient value to be worth their additional cost.

## Supplemental Material

Comparability of Objective Structured Clinical Examinations (OSCEs) and Written Tests for Assessing Medical School Students’ Competencies: A Scoping Review Click here for additional data file. Comparability of Objective Structured Clinical Examinations (OSCEs) and Written Tests for Assessing Medical School Students’ Competencies: A Scoping Review by Oswin Chang, Anne M Holbrook, Simran Lohit, Jiawen Deng, Janice Xu, Munil Lee, Alan Cheng in Evaluation & the Health Professions

Supplemental Material - Comparability of Objective Structured Clinical Examinations (OSCEs) and Written Tests for Assessing Medical School Students’ Competencies: A Scoping Review Click here for additional data file. Supplemental Material for Comparability of Objective Structured Clinical Examinations (OSCEs) and Written Tests for Assessing Medical School Students’ Competencies: A Scoping Review by Oswin Chang, Anne M Holbrook, Simran Lohit, Jiawen Deng, Janice Xu, Munil Lee, Alan Cheng in Evaluation & the Health Professions
