# Supplementary material for: Comparability of Objective Structured Clinical Examinations (OSCEs) and Written Tests for Assessing Medical School Students’ Competencies: A Scoping Review
Source: Eval Health Prof. 2023 Mar 23;46(3):213–24. doi: 10.1177/01632787231165797 (PMC10443966; doi:10.1177/01632787231165797)
Supplement: Supplemental Material - Comparability of Objective Structured Clinical Examinations (OSCEs) and Written Tests for Assessing Medical School Students’ Competencies: A Scoping Review [file sj-pdf-2-ehp-10.1177_01632787231165797.pdf]

Supplemental Appendix 1. Search Strategies

A. *Search strategy for MEDLINE*

1. CanMEDS.mp.
2. Communication/
3. exp *Professional Competence/
4. Cooperative Behavior/
5. *Leadership/
6. *Patient Advocacy/
7. *Research Personnel/
8. professional*.ti,kf.
9. communicat*.ti,kf.
10. collaborat*.ti,kf.
11. leader*.ti,kf.
12. advocat*.ti,kf.
13. scholar?.ti,kf.
14. expert?.ti,kf.
15. ((clinical* OR medical* OR physician* OR professional*) ADJ2 competenc*).ti,kf.
16. or/1-15

---

17. (objective* ADJ3 clinical* ADJ3 exam*).ti,kf.
18. (objective structured clinical exam* OR OSCE?).mp.
19. or/17,18

---

20. *Educational Measurement/
21. exp *Academic Performance/
22. Test Taking Skills/
23. (test? OR testing OR exam* OR evaluat* OR assessment*).tw,kf.
24. (multiplechoice? OR multiple-choice? OR written).mp.
25. or/20-24

---

26. (virtual* OR online* OR on-line* OR web-base* OR digital* OR remote* OR tele*).ti,kf.
27. Education, distance/
28. or/26,27

---

29. and/16,19,25 **(Research Question 1)**
30. and/19,28
31. Limit 30 to yr="2000-Current" **(Research Question 2)**

B. Search strategy for EMBASE

1. CanMEDS.mp.
2. Communication/
3. exp *Professional Competence/
4. Cooperative Behavior/
5. *Leadership/
6. *Patient Advocacy/
7. *Research Personnel/
8. professional*.ti,kw.
9. communicat*.ti,kw.
10. collaborat*.ti,kw.
11. leader*.ti,kw.
12. advocat*.ti,kw.
13. scholar?.ti,kw.
14. expert?.ti,kw.
15. ((clinical* OR medical* OR physician* OR professional*) ADJ2 competenc*).ti,kw.
16. or/1-15

---

17. (objective* ADJ3 clinical* ADJ3 exam*).ti,kw.
18. (objective structured clinical exam* OR OSCE?).mp.
19. or/17,18

---

20. *Educational Measurement/
21. exp *Academic Performance/
22. Test Taking Skills/
23. (test? OR testing OR exam* OR evaluat* OR assessment*).tw,kw.
24. (multiplechoice? OR multiple-choice? OR written).mp.
25. or/20-24

---

26. (virtual* OR online* OR on-line* OR web-base* OR digital* OR remote* OR tele*).ti,kw.
27. Education, distance/ OR educational measurement.mp.
28. or/26,27

---

29. and/16,19,25 **(Research Question 1)**
30. and/19,28
31. Limit 30 to yr="2000-Current" **(Research Question 2)**

Supplemental Appendix 2. Screening Exclusion Criteria

A. *Research Question #1 Title/Abstract Screening Exclusion Criteria*

1. Is the study population health professional trainees (e.g., students or residents), excluding veterinary trainees?
- Yes -> Continue
- No -> Exclude

2. Does the study supply primary data?
- Yes -> Continue
- No -> Exclude

3. Does the study compare OSCEs with written/typed testing methods (e.g., multiple-choice questions, short/long form answers)?
- Yes -> Continue
- No -> Exclude

4. Does the study compare testing methods in their ability to assess one or more CanMEDS domains (Medical Expert, Communicator, Collaborator, Leader, Health Advocate, Scholar, Professional)?*
- Yes -> Continue
- No -> Exclude

**Outcomes do not have to be expressly stated as being part of the CanMEDS framework. For example, a study looking at teamwork would be classified under Collaborator.*

5. Is the study written in English/is there an English summary which provides the necessary information?
- Yes -> Continue
- No -> Exclude

B. *Research Question #2 Title/Abstract Screening Exclusion Criteria*

1. Is the study population health professional trainees (e.g., students or residents), excluding veterinary trainees?
- Yes -> Continue
- No -> Exclude

2. Does the study supply primary data?

- Yes -> Continue
- No -> Exclude

3. Does the study compare in-person OSCEs with online OSCEs?
- Yes -> Continue
- No -> Exclude

4. Does the study compare testing methods in their ability to assess the CanMEDS roles, logistics, feasibility, resource intensity, or other aspects between the two OSCE formats?
- Yes -> Continue
- No -> Exclude

5. Is the study written in English/is there an English summary which provides the necessary information?
- Yes -> Continue
- No -> Exclude

Supplemental Appendix 3. Included Article Characteristics for Written Tests vs. OSCEs

Reference	Country	Type of health professional trainee	Written assessment(s) used	Competency domain(s) assessed	Strength of correlations (overall score comparisons and competency domain score comparisons)*
Andrades et al., 2017	Pakistan	Medicine	MCQ	Medical Expert, Communicator, Professional	Low, Medium
Auewarakul et al., 2005	Thailand	Medicine	MCQ	Medical Expert	Medium
Butler et al., 2017	USA	Medicine	MCQ	Medical Expert, Communicator, Professional	Low
Chibnall & Blaskiewicz, 2008	USA	Medicine	MCQ	Medical Expert, Communicator	NR
Couto et al., 2019	Brazil	Medicine	MCQ	Medical Expert	Low
Dennehy et al., 2008	USA	Dentistry	MCQ	Medical Expert, Communicator	NR
Dong et al., 2014	USA	Medicine	MCQ	Medical Expert, Communicator	Low
Dong et al., 2017	USA	Medicine	MCQ	Medical Expert, Professional	Low
Eftekhar et al., 2012	Iran	Medicine	MCQ	Medical Expert, Communicator, Professional	Low, Medium
Gillette et al., 2017	USA	Pharmacy	Short answer, long answer/essay	Communicator, Professional	Low

Gilson et al., 1998	USA	Medicine	MCQ	Medical Expert, Communicator	Low
Hull et al., 1995	USA	Medicine	MCQ	Medical Expert	Low
Huwendiek et al., 2017	Germany	Medicine	MCQ, Short answer, long answer/essay	Medical Expert, Communicator	Medium
Jameel et al., 2015	Pakistan	Medicine	Short answer	Communicator, Professional	Low, Medium
Kelly et al., 2013	Ireland	Medicine	MCQ	Medical Expert, Communicator	Low
Nuovo et al., 2006	USA	Medicine	Long answer/essay	Medical Expert, Communicator	NR
Schleicher et al., 2017	Germany	Medicine	MCQ	Medical Expert	Low
Schoeman & Chandratilake, 2012	Britain	Medicine	MCQ, Short answer	NR	Low, Medium
Schwartz et al., 1995	USA	Medicine	MCQ	Medical Expert, Communicator	Medium
Simon et al., 2002	USA	Medicine	MCQ	Medical Expert, Communicator, Professional	Low, Medium
Simon et al., 2007	USA	Medicine	MCQ	Medical Expert, Communicator, Professional	Low

Abbreviations: OSCE, Objective Structured Clinical Examination; MCQ, multiple-choice question; NR, not reported
*low = <0.40, medium = 0.40-0.79, high = >0.79

Supplemental Appendix 4. Included Article Characteristics for In-Person vs. Online OSCEs

Reference	Country	Type of health professional trainee	Competency domains assessed	Overall score comparisons between in-person and online OSCEs
Arrogante et al., 2021	Spain	Nursing	Medical Expert, Communicator, Collaborator	No difference
Biolik et al., 2018	Germany	Medicine	Medical Expert	No difference
Boardman et al., 2021	USA	Medicine	Communicator, Health Advocate, Professional	NR
Darr et al., 2021	USA	Pharmacy	Medical Expert	No difference
Farrell et al., 2021	USA	Medicine	Medical Expert, Communicator	No difference
Hsia et al., 2021	USA	Pharmacy	Communicator	NR
Lara et al., 2020	USA	Medicine	NR	No difference
Martinez et al., 2020	USA	Medicine	Medical Expert, Communicator	No difference
Nackman et al., 2006	USA	Medicine	Medical Expert	No difference
Novack et al., 2002	USA	Medicine	Medical Expert, Communicator, Collaborator, Health Advocate, Professional	NR
Oliven et al., 2011	Israel	Medicine	Medical Expert, Communicator	No difference
Scoular et al., 2021	USA	Pharmacy	Communicator, Professional	Higher in online
Sihombing et al., 2021	Indonesia	Medicine	Medical Expert, Communicator	No difference, higher in online

Abbreviations: OSCE, Objective Structured Clinical Examination; NR, not reported
